# Selective Deposition of Charged Droplets for Programmable and Rewritable Printing of Patterned Microstructure Arrays

**DOI:** 10.1002/advs.202522210

**Published:** 2026-02-24

**Authors:** Yuechen Pei, Li Wang, Zhaofa Zhang, Yulin Zheng, Tianxue Yin, Jiaye Chen, Juntao Zhang, Yujie Qi, Bingheng Lu

**Affiliations:** ^1^ The State Key Laboratory for Manufacturing Systems Engineering Xi'an Jiaotong University Xi'an China; ^2^ School of Mechanical Engineering Xi'an Jiaotong University Xi'an China; ^3^ National Innovation Institute of Additive Manufacturing Xi'an China

**Keywords:** electrospray, information encryption, patterned microstructure arrays

## Abstract

Patterned microstructure arrays are widely used in flexible electronics, optics, and biosensing, yet fabrication still relies on costly, process‐intensive methods (e.g., photolithography) with limited reconfigurability. In this work, we introduce a method for the selective deposition of charged droplets via residual‐charge‐induced electric field control (SDREC), enabling microstructure‐array patterning with a resolution of 20 µm through the controlled assembly of charged microdroplets on silk fibroin surfaces. As a proof of concept, a programmable 5×5 pixel array was fabricated, demonstrating reversibility through erasure and rewriting. By transferring the patterned microstructures onto a shape memory polymer, we further achieved information storage and optical encryption/decryption. In addition, pre‐deposition of silver nanoparticles within the patterned areas enabled the fabrication of silver electrodes. Overall, the SDREC strategy offers advantages such as high resolution, structural reconfigurability, and multifunctionality, providing a promising approach for the rapid construction of high‐precision microstructured devices.

## Graphical abstract

A residual‐charge‐induced electric field control (SDREC) strategy enables the fabrication of patterned microstructure arrays with a resolution of 20 µm via the selective deposition of charged droplets. The resulting arrays exhibit reversible writing/erasing, optical encryption, and selective silver electrode formation, offering a versatile route to multifunctional microstructured devices.

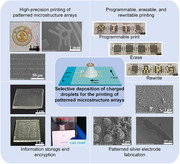

## 1 Introduction

Numerous biological systems feature distinctive functional microstructure arrays that have evolved over long timescales and confer exceptional properties [1, 2]. Observation and emulation of such microstructure arrays have enabled the development of innovative materials and technologies, markedly enhancing performance and efficiency in industrial and biomedical contexts [3, 4, 5, 6, 7, 8]. Representative examples include the superhydrophobic architecture of lotus leaves [9, 10, 11], which has inspired water‐repellent material design, and the micro‐ and nanostructures of gecko feet [12, 13, 14, 15, 16], which have motivated high‐adhesion materials. Collectively, these examples underscore the substantial potential of microstructure‐guided biomimetics: by emulating natural architectures, one can achieve functions characterized by high efficiency, reduced energy consumption, and smart behavior.

Patterned microstructure arrays play indispensable roles across diverse fields. Patterning at the macro‐ and microscale endows materials with tailored optical [17, 18, 19], electrical [20, 21, 22], and mechanical properties [23], as well as controlled surface wettability [24, 25, 26]. To realize biomimetic microstructures in practical applications, the choice of fabrication methodology is therefore critical. Currently, microstructure fabrication primarily relies on high‐precision patterning techniques such as photolithography [27, 28], electron‐beam lithography (EBL) [29, 30, 31], and nanoimprint lithography (NIL) [32, 33, 34]. In photolithography, light defines features in a photoresist, which are subsequently transferred to the target substrate by etching or via deposition and lift‐off. Photolithography offers excellent resolution and reproducibility; however, it typically requires cleanroom infrastructure, photomasks, and multistep processing (e.g., resist coating, exposure, development, and pattern transfer), which increases the cost and limits rapid iteration. EBL directly writes micro/nanoscale features in a resist with an electron beam, enabling ultrahigh resolution but suffering from low throughput and high cost, which confines its use largely to small‐scale laboratory prototyping. NIL provides a more cost‐effective route by mechanically imprinting pre‐designed nanopatterns onto a substrate, offering good versatility and process efficiency. Nevertheless, NIL faces practical constraints: imprint molds wear over time and have limited lifetimes, and switching to different patterns requires fabricating or replacing physical stamps. These factors make rapid pattern reconfiguration time‐consuming and costly. Overall, these established approaches involve trade‐offs among resolution, infrastructure cost, and pattern reconfigurability, as summarized in Table S1.

Recently, we introduced a fabrication approach for microstructure arrays based on the controlled assembly of charged microdroplets (CACM) [35]. The method operates through two coupled positive‐feedback loops. First, charged droplets generated by electrospraying partially dissolve the silk‐fibroin substrate; as solvent evaporates, capillary flow drives ring‐like deposits (coffee‐ring effect). Second, after evaporation, residual charges retained on the substrate—together with the emerging surface relief—create an electrostatic‐lens effect that biases subsequent charged droplets to deposit within local concavities. Deposition into these concavities deepens the pits, which further strengthens the electrostatic focusing and, in turn, increases the likelihood of additional droplet deposition at the same sites. Through this self‐reinforcing process, thin films with surface honeycomb‐like pore arrays are obtained. Notably, by tuning key electrospray parameters (e.g., applied voltage and flow rate), CACM enables controllable fabrication of pore‐type microstructures with pore diameters ranging from 0.9 to 3.4 µm. However, unlike photolithography and other patterning methods, this process cannot yet fabricate patterned microstructure arrays.

**FIGURE 1:**
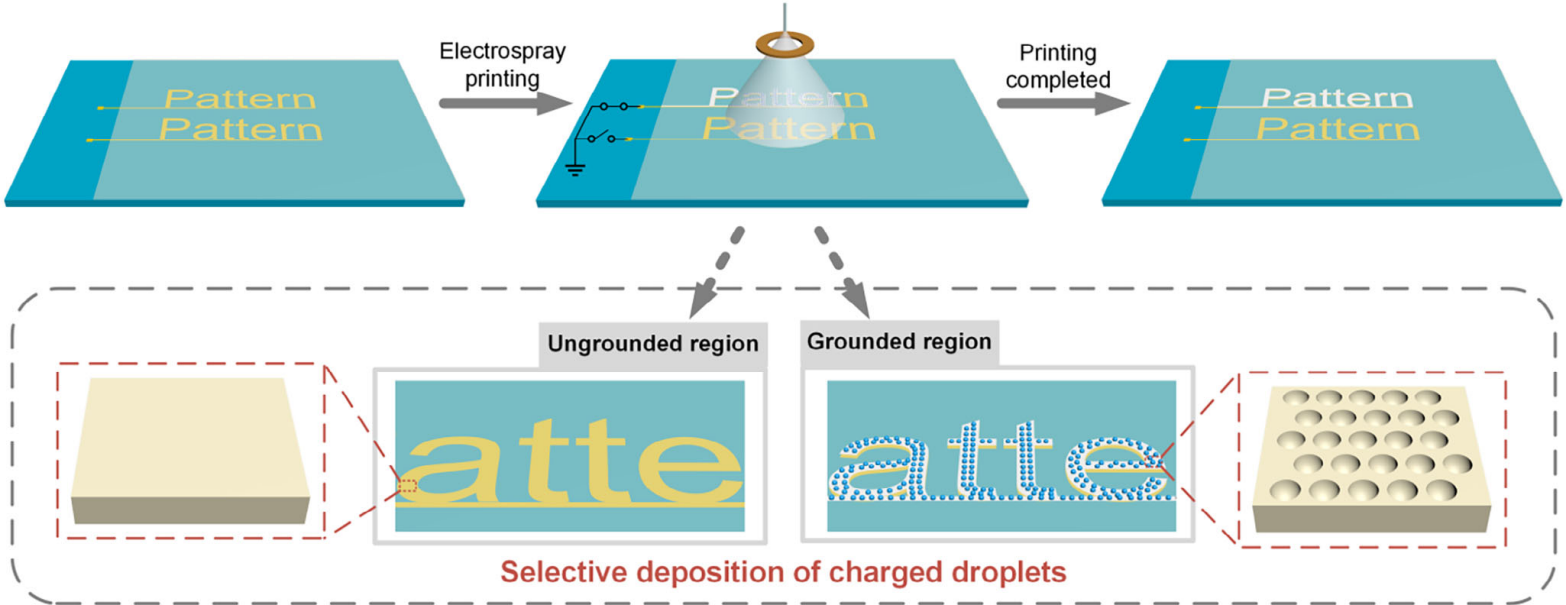
Schematic illustration of the selective deposition of charged droplets via residual‐charge‐induced electric field control (SDREC). This method enables programmable microstructure patterning through controlled electrospray deposition. By switching the grounding state of the electrode beneath the substrate, the residual charge‐induced electric field can be spatially modulated, guiding the selective deposition of charged droplets onto targeted areas. Subsequent processing using the CACM method results in the formation of microstructures.

Here, we propose a method for the selective deposition of charged droplets via residual‐charge‐induced electric field control (SDREC, Figure 1). By switching the electrodes between grounded and ungrounded states, the residual‐charge–induced electric field at the substrate surface is modulated to steer electrosprayed charged‐droplet ensembles toward designated regions, thereby enabling selective deposition. Importantly, the forming material and solvent system used in this work are identical to those in our previous CACM study [35], and thus the established CACM morphology–parameter relationships remain applicable. In the present study, we deliberately operate within a stable CACM regime and fix the electrospray parameters (e.g., voltage and flow rate), so that the observed spatial selectivity and patterned formation can be attributed to SDREC‐enabled field modulation. Coupling this field control with the subsequent CACM process yields microstructured patterns with a minimum feature size of 20 µm. Leveraging a pixelated dot‐matrix electrode together with the aqueous solubility of silk fibroin enables programmable, erasable, and rewritable microstructure patterning. Replication of these microstructures in a shape‐memory polymer enables information storage and encryption: the encoded patterns remain concealed within the film and become readable upon thermal stimulation. Furthermore, pre‐seeding silver nanoparticles within the microstructured regions facilitates site‐selective silver growth, producing patterned silver electrodes.

## 2 Results

### 2.1 Selective Deposition of Charged Droplets via Residual‐Charge‐Induced Electric Field Control

**FIGURE 2:**
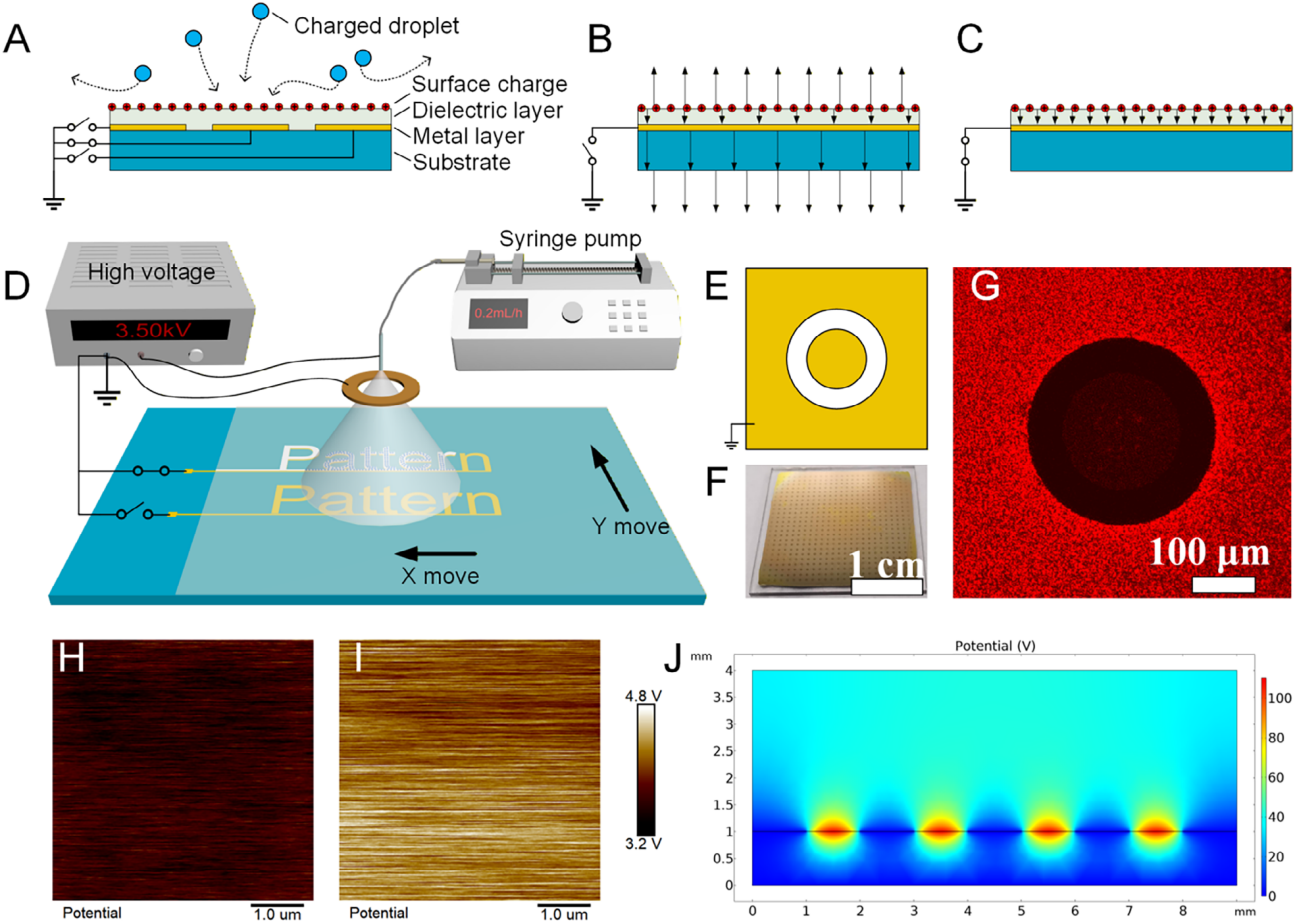
Working mechanism and experimental validation of the SDREC system. (A) Schematic structure of the SDREC panel, composed of a bottom quartz glass substrate, a middle switchable gold electrode layer, and a top insulating silk fibroin film. The quartz glass minimizes induced charges due to its low ionic doping level. The silk fibroin layer serves both to isolate residual charges and to mold the honeycomb microstructures. (B) Electrostatic model for a uniformly charged surface (surface charge density *σ*): with the metal layer ungrounded under electrostatic equilibrium, the field above the panel is *E* = *σ*/(2ε) and points upward, so incoming charged droplets are Coulombically repelled and inhibited from depositing. (C) With the metal layer grounded, the external field above the panel cancels (*E* ≈ 0), enabling facile droplet deposition. (D) Patterned microstructure printing system based on SDREC (see also Figure S1), consisting of: a liquid delivery unit, a high‐voltage power supply, the SDREC panel to modulate droplet deposition via charge field control, a grounded copper ring for Taylor cone stabilization and excess droplet collection, and an XY‐stage for area‐wide patterning. (E) Design of the circular‐island‐shaped electrode used to investigate field‐guided deposition. (F) Fabricated SDREC panel corresponding to (E). (G) Rhodamine B/ethanol electrospray visualization of droplet landing: grounded‐metal regions appear dark red (high deposition), whereas floating‐metal or metal‐free regions appear light red (low deposition), consistent with the field‐distribution analysis in (B,C). (H, I) PeakForce KPFM surface potential maps recorded above the silk fibroin surface after uniform charging by electrospraying ethanol (electrodes initially ungrounded). Scans were taken far from electrode edges. (H) Above a grounded Au‐electrode region: 3.64 ± 0.12 V. (I) Above a region with no grounded electrode underneath: 4.23 ± 0.18 V. (J) Finite‐element electrostatic simulation of the quartz/electrode/silk stack (1 mm quartz, 3‐µm silk; ε_r_(quartz) = 3.8, ε_r_(silk) = 6.1) with a uniform surface charge density of 1 × 10^−^^5^ C m^−^^2^ on the silk surface, showing lower surface potential above grounded‐electrode regions, consistent with (H,I).

During electrohydrodynamic (EHD) printing—particularly in electrospraying—charged droplets that land on the substrate and evaporate leave behind a portion of charge on the substrate surface, referred to as residual charge [36, 37, 38]. Residual charge can exert Coulombic repulsion on subsequently arriving charged droplets, deflecting their trajectories and thereby degrading print quality and increasing positional errors; if sufficiently accumulated, it may even perturb the stability of the Taylor cone and thus compromise the overall stability of the printing process [39, 40, 41, 42]. Notably, recent studies have shown that depositing and/or reconfiguring surface charges on dielectric films or conductive/nonconductive patterned surfaces can generate strong local electric‐field gradients, enabling programmable and reconfigurable droplet behaviors [43, 44]. Here, we propose leveraging residual charge to generate a patterned electric field. The proposed SDREC panel comprises three layers (Figure 2A). The bottom layer is a substrate made of an insulating material (quartz glass). Compared with conventional soda–lime glass, quartz contains far fewer dopant ions, markedly reducing induced charge and improving the fidelity of the patterned electric field produced by the panel. The top layer is an insulating silk fibroin film that serves two functions: (i) it electrically isolates incoming charged droplets from the metal layer to preserve residual charge, and (ii) it acts as the molding material for the honeycomb porous microstructures. Sandwiched between them is a metal layer (a gold thin film) whose connection to ground can be switched. To elucidate how grounding the metal layer affects the electric‐field distribution generated by residual charge, we analyze two cases. Assuming an idealized planar geometry with a uniform surface‐charge distribution with density σ and taking the permittivity of air as ε: when the metal layer is ungrounded and the system is in electrostatic equilibrium (Figure 2B), the electric field above the SDREC panel has magnitude *σ*/(2ε) and points upward, so charged droplets experience Coulombic repulsion and are inhibited from depositing on the substrate; when the metal layer is grounded and electrostatic equilibrium is established (Figure 2C), the electric field above the panel vanishes (*E* = 0), enabling charged droplets to deposit readily onto the substrate. This behavior arises because grounding renders the metal layer equipotential with the earth, allowing the induced charge on its lower surface to disperse over the much larger terrestrial surface. The detailed derivation is provided in Note and Figure S1. Notably, the *E* = 0 result corresponds to this idealized limit; in the practical device geometry, the external field above grounded regions is reduced but may not vanish everywhere because of finite‐size and fringing effects.

We designed a patterned microstructure array printing system based on SDREC. As illustrated in Figure 2D and Figure S2, the system comprises five modules: a fluid delivery unit, a high‐voltage power supply, an SDREC panel, a grounded copper ring, and a motorized X–Y two‐axis translation stage. The fluid delivery unit consists of a 28G Musashi needle (serving as the nozzle), a 1 mL syringe, and a syringe pump, enabling precise flow‐rate control during printing. The high‐voltage supply provides the stable operating voltage required for electrospraying and establishes the electric field between the nozzle and the grounded copper ring. The SDREC panel enables the selective deposition of charged droplets and yields honeycomb‐like porous microstructures within the deposition zones. The grounded copper ring (inner diameter 14 mm, outer diameter 24 mm, thickness 2 mm) serves two functions: (i) it electrostatically shields the patterned field generated by the SDREC panel from perturbing the Taylor cone at the nozzle, thereby stabilizing the electrospray; and (ii) it attracts and collects the fraction of charged droplets that are repelled by the SDREC panel. The motorized X–Y translation stage permits electrospray printing over the entire active area of the SDREC panel, ensuring complete coverage during pattern formation.

To validate the operating principle of SDREC, we designed an electrode layout featuring a central circular island (Figure 2E) and fabricated a corresponding SDREC panel (Figure 2F). In the experiments, a rhodamine B (RhB) ethanol solution was electrosprayed onto the SDREC surface to visualize droplet landing. The results show that regions where the metal layer is grounded appear dark red, indicating that most droplets deposit in these areas; by contrast, regions with an ungrounded metal layer or without a metal layer appear light red, indicating that only a small fraction of droplets deposit under the influence of residual charge (Figure 2G). These observations are consistent with the field‐distribution analysis: grounding the metal layer effectively cancels the local residual‐charge‐induced field and reduces electrostatic repulsion on incoming charged droplets, thereby promoting deposition, whereas ungrounded or metal‐free regions retain the residual‐charge field, produce significant electrostatic repulsion, and suppress deposition.

To directly verify the residual‐charge‐induced field modulation underlying SDREC, we combined Kelvin probe force microscopy (KPFM) measurements with finite‐element electrostatic simulations. In the experiment, charges were first introduced uniformly onto the SDREC panel by electrospraying pure ethanol while keeping all electrodes floating (ungrounded). PeakForce KPFM mapping was then performed with the panel electrically referenced to ground, and the scanned regions were intentionally selected far from electrode edges to minimize boundary artifacts. Importantly, the KPFM signal was recorded above the top surface of the silk fibroin dielectric. As shown in Figure 2H,I, the surface potential measured above the silk fibroin surface over a grounded Au‐electrode region is 3.64 ± 0.12 V, which is lower than that measured above the silk fibroin surface over a region with no grounded electrode underneath (4.23 ± 0.18 V). This observation provides direct evidence that the grounding state of the underlying electrode modulates the charge‐induced surface potential at the dielectric top surface and thus alters the effective near‐surface field experienced by incoming charged droplets.

To further support this interpretation, we established a finite‐element model based on the experimental stack (1‐mm quartz / patterned electrodes / 3‐µm silk fibroin). The relative permittivities were set to ε_r_ = 3.8 (quartz) [45] and ε_r_ = 6.1 (silk fibroin) [46], and a uniform surface charge density of 1 × 10^−^^5^ C m^−^^2^ was applied on the silk fibroin surface. With the quartz bottom and patterned electrodes grounded, the simulation (Figure 2J) predicts a lower surface potential above grounded‐electrode regions than above ungrounded regions, consistent with the KPFM results. Together, these results substantiate the residual‐charge‐induced field modulation mechanism employed in SDREC.

**FIGURE 3:**
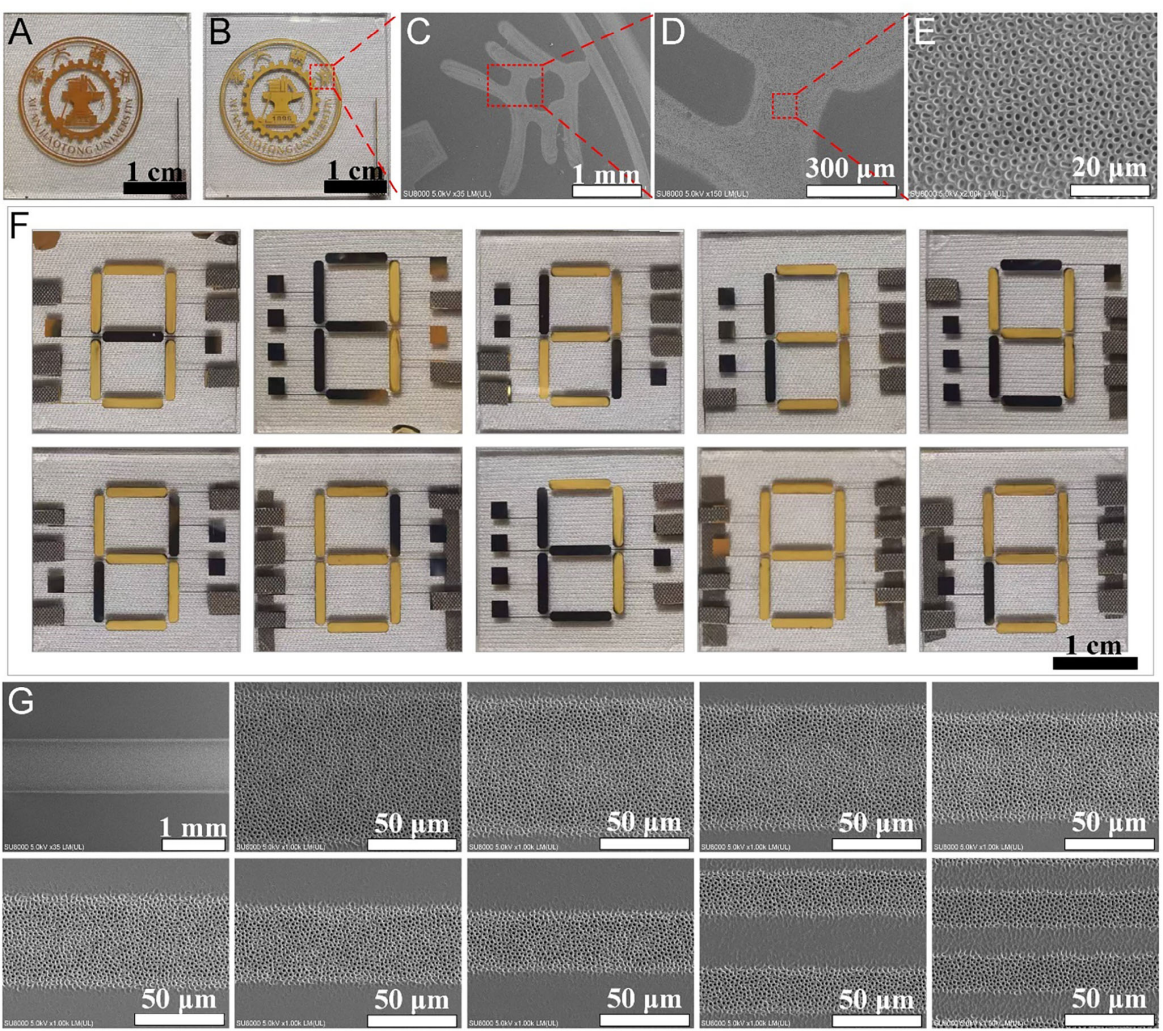
Microstructure patterning via SDREC. (A) SDREC panel whose electrode layer is patterned with the Xi'an Jiaotong University (XJTU) emblem. (B) Honeycomb‐like porous microstructures produced by selective deposition of charged droplets and subsequent CACM on the silk‐fibroin overlayer, reproducing the XJTU emblem. (C–E) Higher‐magnification SEM images of representative regions in (B). (F) Seven independently addressable line segments (seven‐segment architecture) capable of displaying the digits 0–9; for example, rendering “0” grounds all segments except the central one. (G) Resolution test using line‐shaped electrode patterns: SDREC enables line arrays with feature widths spanning 1 mm down to 20 µm (representative widths: 100, 90, 80, 70, 60, 50, 40, and 30 µm).

Using SDREC, we designed electrode patterns with various geometries. When the metal electrode layer is grounded, charged droplets continuously deposit within the grounded regions. Consistent with our previous work, the droplets undergo Controlled Assembly of Charged Microdroplets on the silk‐fibroin surface, forming micrometer‐scale honeycomb‐like porous structures. Figure 3A shows an SDREC panel whose electrode layer is patterned with the emblem of Xi'an Jiaotong University (XJTU). Figure 3B displays the honeycomb‐like porous microstructures produced by the selective deposition of charged droplets on the silk‐fibroin overlayer, reproducing the XJTU emblem; scanning electron microscopy (SEM) images of selected regions are provided in Figure 3C–E. Figure 3F presents a pattern consisting of seven independently addressable line pixels (a seven‐segment architecture) capable of displaying the digits 0–9. For example, to render the numeral “0,” all segments except the central one are grounded. To evaluate the printing resolution of SDREC, we further designed line‐shaped electrode patterns with different widths. As shown in Figure 3G, SDREC enables the fabrication of line‐array patterns with feature widths spanning 1 mm down to 20 µm, with representative widths of 100, 90, 80, 70, 60, 50, 40, and 30 µm.

### 2.2 Programmable, Erasable, and Rewritable Fabrication of Patterned Microstructure Arrays

**FIGURE 4:**
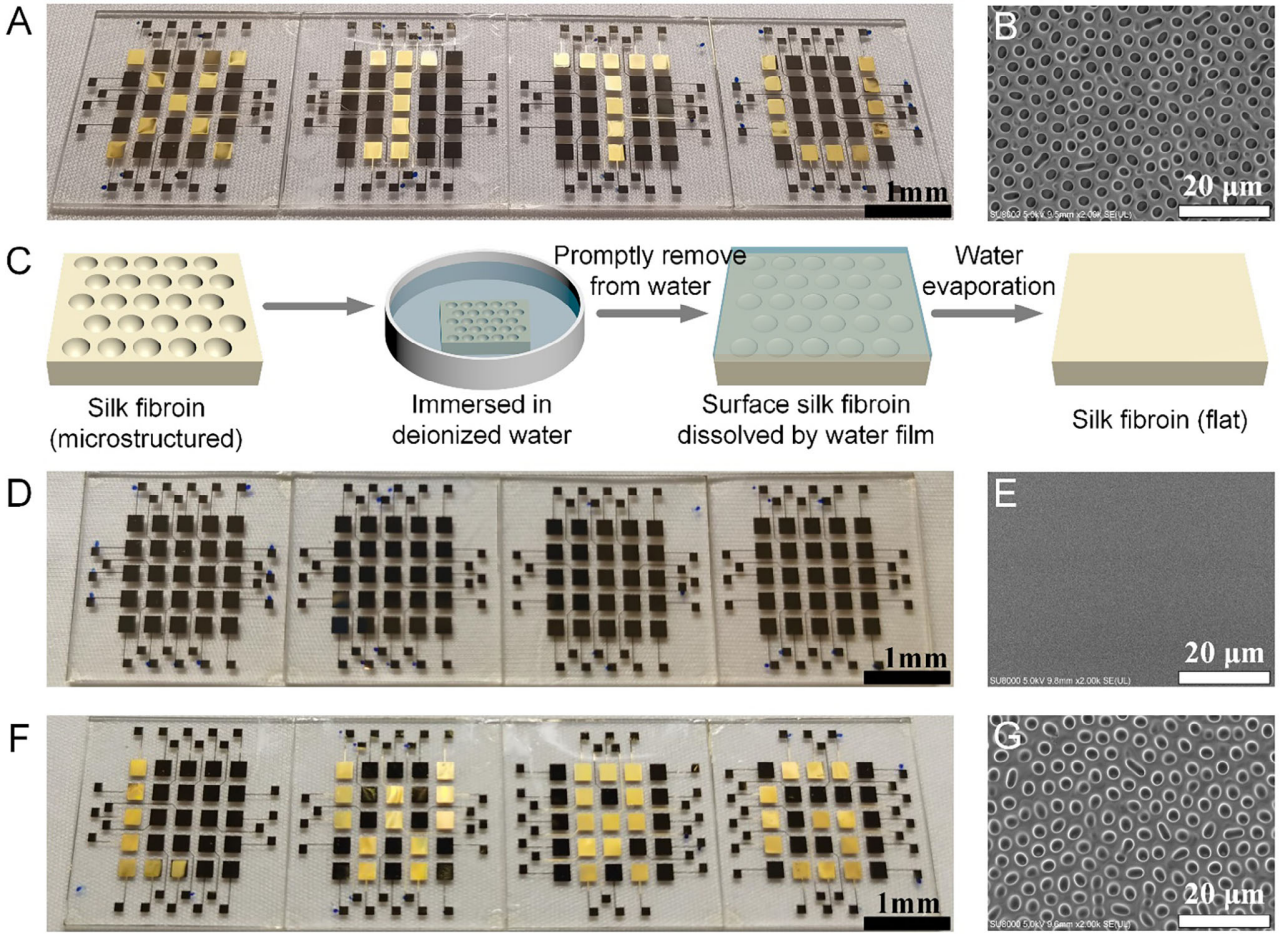
Programmable, erasable, and rewritable fabrication of patterned microstructures. (A) Proof‐of‐concept 5×5 pixelated microelectrode array; grounding selected pixels writes the four letters “XJTU” (Xi'an Jiaotong University). (B) SEM images of the resulting honeycomb‐like porous microstructures on silk fibroin reproducing XJTU. (C) Erasure procedure: rapid dip in deionized water, immediate withdrawal, and gentle removal of droplets; the residual water film dissolves the microstructures, and the pattern vanishes as the water evaporates. (D) SDREC panel after erasure (optical image), showing loss of the visible pattern. (E) SEM confirming a feature‐free, planar surface after erasure. (F) Rewriting on the same substrate by grounding a different pixel set to form “LWRG” (Li Wang's Research Group). (G) SEM of the rewritten microstructures, closely matching the initial print and demonstrating reversible write–erase–rewrite capability.

To overcome the limited applicability imposed by fixed‐shape electrode layouts in microstructure patterning, we further introduce a programmable potential‐gating strategy based on a pixelated dot array. By employing a pixelated microelectrode array on the substrate and toggling circuit connections to dynamically modulate local potentials, charged droplets can be precisely guided during electrospraying to selectively deposit in designated regions, thereby constructing target microstructural patterns. As a proof of concept, we demonstrate programmable patterning using a 5×5 dot‐pixel array. As shown in Figure 4A, grounding different pixel electrodes writes four letters “XJTU” (the abbreviation of Xi'an Jiaotong University); the corresponding microstructural morphology is presented as SEM images in Figure 4B. Notably, the patterned microstructures are not only created on demand but are also erasable and rewritable. Specifically (Figure 4C and Movie S1), the patterned sample is rapidly dipped into deionized water and immediately withdrawn; after gently flicking off droplets, a thin residual water film permeates and dissolves the microstructural material. As the water evaporates, the original pattern gradually vanishes and the substrate surface returns to a flat state. Figure 4D shows the SDREC panel after erasure, and Figure 4E provides SEM images confirming a feature‐free, planar surface. This procedure demonstrates excellent erasability and requires neither complex operations nor external energy input, relying solely on mild liquid contact and evaporation. After erasure, the sample is reintroduced into the SDREC printing system; by grounding a new set of pixel electrodes, a new microstructural pattern is reconstructed on the same substrate. As shown in Figure 4F and Movie S2, the four letters “LWRG” (the abbreviation of Li Wang's Research Group) are successfully rewritten. The morphology of the rewritten microstructures (Figure 4G) closely resembles that of the initial print, indicating that the substrate retains robust formability after the erasure process. To examine whether the DI‐water erasure process introduces any unintended surface damage to the underlying panel, we performed a dedicated morphological assessment using an SDREC panel in which an Au electrode largely covers the quartz substrate and the surface was coated with a silk fibroin film. The panel surface was divided into nine regions (Regions 1–9), and one random location in each region was characterized by laser confocal microscopy before and after erasure (Figure S3). The areal roughness (*S*a) changed only slightly from 0.0176 ± 0.0062 µm (before erasure) to 0.0221 ± 0.0050 µm (after erasure) (*n* = 9), and no apparent surface damage features (e.g., cracking, delamination, or severe roughening) were observed. These results indicate that the DI‐water erasure step can remove the silk fibroin porous microstructures without causing observable surface structural damage to the panel under our experimental conditions.

### 2.3 Information Storage and Encryption

In applications such as information security and anti‐counterfeiting, controllable revelation and concealment of patterns are a key route to encryption [47, 48]. Compared with dye‐based or electronic identifiers, structure‐based information encryption relies on light–microstructure interactions at the surface, offering advantages of no continuous power requirement, reversible response, and high environmental stability [49, 50]. As shown in Figure S4, we first construct patterned microstructures via SDREC and then perform two‐step polydimethylsiloxane (PDMS) replica molding to obtain intermediate positive‐ and negative‐relief molds. Finally, the target microstructures are fully transferred—as a positive (protruding) relief—onto a shape memory polymer (SMP) surface, achieving high‐fidelity pattern replication with functional integration.

**FIGURE 5:**
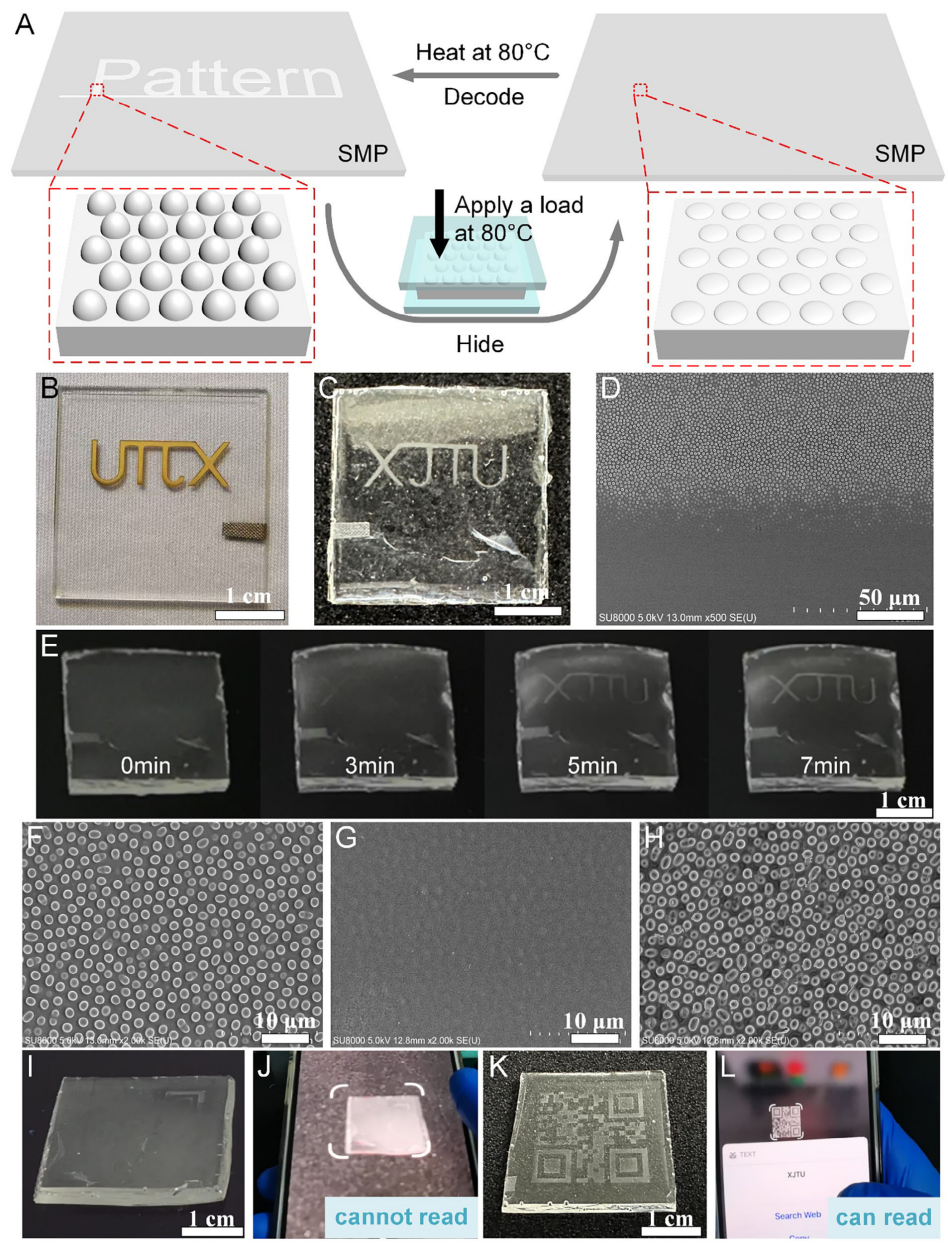
Information storage and encryption using SMP microstructures replicated from SDREC patterns. (A) Thermal encryption/decryption workflow: heating to 80°C under mechanical load flattens the microstructures (encryption); cooling under load fixes the flattened state; reheating to 80°C without load restores the microstructures (decryption). (B) Microstructured sample bearing the letters “XJTU” prepared by SDREC. (C) Corresponding SMP sample obtained via two‐step PDMS replica molding (positive/negative molds) and transfer, yielding a positive (protruding) relief. (D) SEM image at a pattern boundary on the SMP, showing sharp contours and high replication fidelity. (E) Time‐lapse optical readout after concealment: on an 80°C hot plate, the pattern reappears within ≈7 min. (F–H) Morphology sequence: initial (visible), flattened/encrypted, and thermally recovered/decrypted, confirming a reversible show–hide–show cycle. (I–L) QR‐code demonstration: encrypted (flattened) state (I) is not machine‐readable (J); decrypted (recovered) state (K) becomes clear and is accurately decoded (L).

As illustrated in Figure S5, the surface microstructures markedly affect the optical appearance of the SMP. Specifically, regions bearing microstructures exhibit strong light scattering, yielding a haze of 75.3%, whereas smooth, structure‐free regions show a haze of only 4.19%. This contrast transforms patterned areas from an otherwise transparent state to a visibly white appearance against a transparent background, enabling clear visual readout. Leveraging this optical behavior, alphanumeric or graphic information can be embedded in the pattern and, together with the reversible thermoresponse of the SMP, used to realize dynamic encryption with controllable visualization. As shown in Figure 5A, heating to 80°C under mechanical load flattens the microstructures; subsequent cooling under load fixes the flattened state, turning the pattern from white to transparent and thereby hiding the information. Reheating to 80°C without load triggers shape‐memory recovery, restoring the microstructures and re‐revealing the pattern (decryption). Figure 5B shows a microstructured sample bearing the letters “XJTU” (abbreviation of Xi'an Jiaotong University) prepared by SDREC; Figure 5C shows the corresponding SMP sample fabricated via the double PDMS molding route. A SEM image of a pattern boundary on the SMP (Figure 5D) reveals sharp contours and high replication fidelity. After concealing the information, placing the SMP on an 80°C hot plate causes the pattern to reappear within ≈7 min (Figure 5E; Movie S3). Figure 5F–H presents the morphology sequence—initial, flattened (encrypted), and thermally recovered—further confirming the reversible show–hide–show cycle. To assess functional pattern recognition, we designed a microstructured pattern encoding a quick response (QR) code. As shown in Movie S4, the QR code is not machine‐readable in the encrypted (flattened) state (Figure 5I,J), but becomes clear and is accurately decoded in the decrypted (recovered) state (Figure 5K,L). The encryption/decryption process is repeatable for at least 10 cycles at 80°C, as evidenced by cycle‐by‐cycle optical images (Figure S6) and confocal morphology tracking (Figure S7), showing consistent disappearance/reappearance of the “XJTU” pattern and reversible flattening/recovery of the microstructures.

### 2.4 Patterned Silver Electrode Fabrication

**FIGURE 6:**
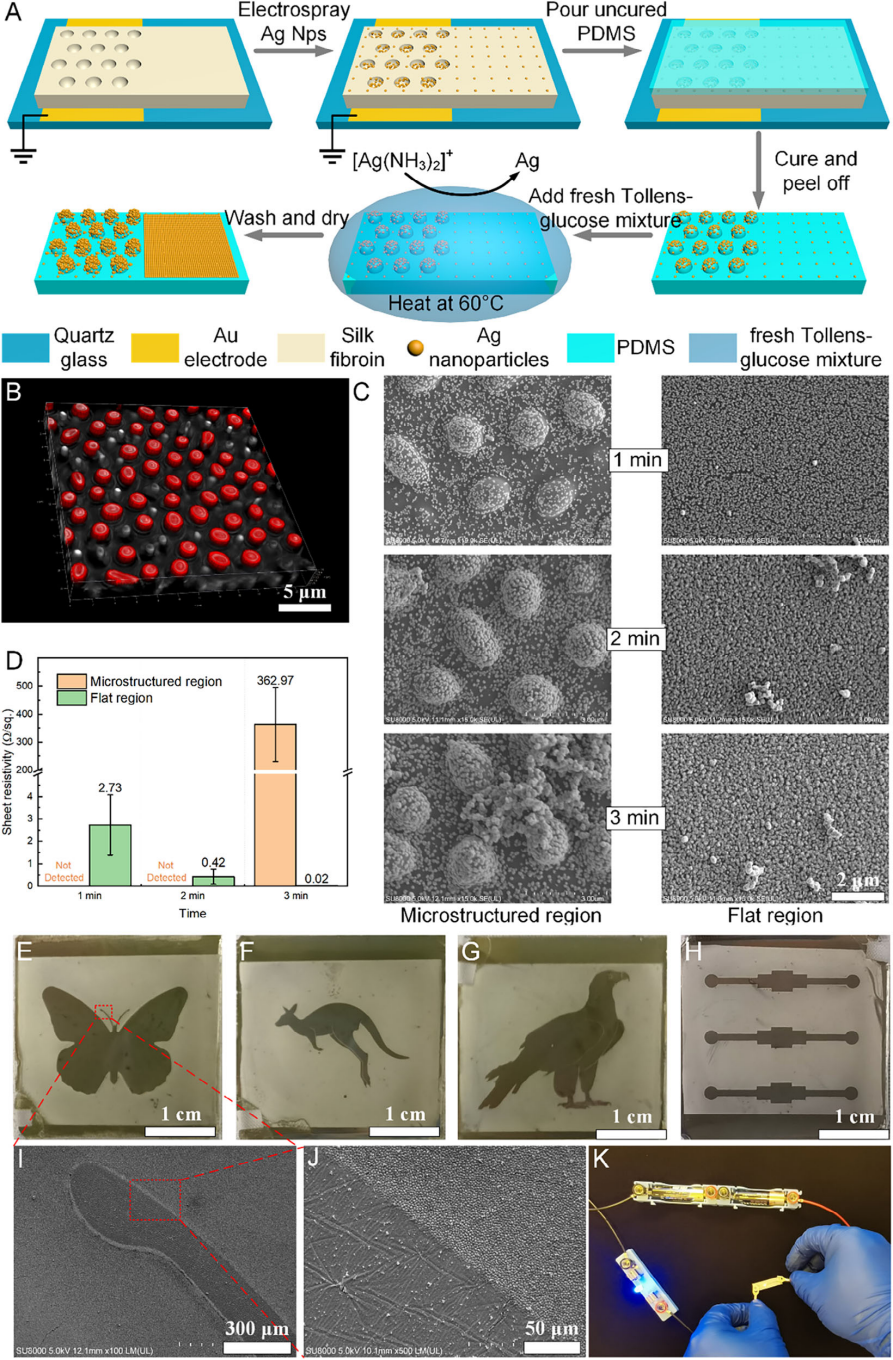
Patterned silver electrode fabrication. (A) Process schematic: electrospray deposition of Ag nanoparticle seeds into microstructured regions (electrostatic lensing), PDMS replication to transfer seeds to protrusion tops, and selective Tollens’ silvering to form conductive Ag patterns. (B) Confocal fluorescence 3D reconstruction after RhB‐doped Ag seeding: PDMS topography (gray) and RhB fluorescence (red) show signal concentrated at protrusion apices, indicating top‐enriched Ag seeds. (C) Selective silver growth during the silver‐mirror reaction: microstructured areas nucleate Ag at tops (island‐like clusters at early times), whereas flat regions develop continuous dense Ag films. (D) Four‐point sheet resistance (ohms sq^−1^): flat regions ≈2.73 ohms sq^−1^ (1 min) and 0.42 ohms sq^−1^ (2 min); microstructured regions initially nonconductive, becoming ≈362.97 ohms sq^−1^ at 3 min as connection occurs. (E–G) Examples of patterned Ag structures (butterfly, kangaroo, eagle). (H) Wire‐shaped Ag electrode. (I,J) Higher‐magnification SEM images of representative regions in (E). (K) Functional demonstration: the Ag electrode integrated in a circuit lights an LED.

Prior studies have shown that silver nanoparticles can act as activators during chemical silvering, providing nucleation sites for metallic Ag deposition [51, 52]. Building on the SDREC‐fabricated microstructures, we introduced an electrospray‐based method to deposit silver nanoparticles as a seed layer selectively within the microstructured regions, thereby defining preferential growth sites for a subsequent silver mirror (Tollens’) reaction. This strategy enables the construction of conductive patterns (Figure 6A).

In our previous work, we found that charged particles or droplets generated by electrospraying can be directionally deposited at the bottoms of concave features via electrostatic lensing [35, 53]. Leveraging this effect, silver nanoparticles were precisely deposited at the bases of the pre‐patterned microstructures. Subsequent polydimethylsiloxane (PDMS) replica molding transferred these bottom‐seeded nanoparticles onto the crests of a newly replicated positive relief, thereby pre‐positioning Ag seeds at the microstructure tops. To verify the spatial distribution of the nanoparticles, we doped the Ag nanoparticle dispersion with rhodamine B (RhB, 0.1 mg mL^−1^) as a fluorescent tracer. After electrospraying this solution onto the microstructured areas and performing PDMS replication, confocal fluorescence 3D reconstructions of the replicated sample (Figure 6B) show the PDMS topography in gray and RhB fluorescence in red. The fluorescence is highly concentrated at the apices of the protrusions, indicating that the electrosprayed silver nanoparticles are enriched at microstructure tops rather than randomly distributed.

As illustrated in Figure 6C, during the chemical silvering reaction, the microstructured regions—owing to the predeposited Ag nanoparticles at their tops—induce spatially selective growth of metallic Ag, whereas the unpatterned flat areas, lacking such seeds, exhibit comparatively uniform deposition. Notably, at early reaction times the Ag on microstructure tops appears as island‐like clusters that are not yet electrically percolated and therefore nonconductive, while the flat regions develop continuous, dense Ag films with good conductivity. As a control (Figure S8), otherwise identical PDMS microstructured samples without preseeded Ag nanoparticles were processed under the same conditions; Ag deposited uniformly across both microstructured and flat regions, confirming the activation role of the preseeded nanoparticles in the silver mirror reaction. To further assess electrical performance, we measured sheet resistance (ohms per square; abbreviated ohms sq^−1^) using the four‐point probe method across different regions (Figure 6D). After 1 and 2 min of reaction, the flat regions showed sheet resistances of approximately 2.73 and 0.42 ohms sq^−1^, respectively, evidencing excellent conductivity, whereas the microstructured regions remained nonconductive with readings beyond the instrument range. With longer reaction times, Ag on the microstructure tops progressively connected; at 3 min, their sheet resistance decreased to ≈362.97 ohms sq^−1^, indicating the onset of conductivity. By contrast, in control samples without Ag seeding, all regions were already conductive at 1–3 min (Figure S9).

These observations define a critical time window for the seeded samples: if the reaction is terminated when the microstructured regions are still nonpercolated while the flat regions have already formed continuous conductive films, patterned conductive Ag can be obtained. Accordingly, we halted the reaction at 2 min, yielding conductive pathways only in the nonstructured areas while the microstructured patterns remained electrically insulating, thus producing a selectively conductive silver pattern. As shown in Figure 6E–G, patterned Ag structures with butterfly, kangaroo, and eagle shapes were successfully fabricated. Figure 6H presents a wire‐shaped Ag pattern. Figure 6I–J shows SEM images of selected regions from Figure 6E. Figure 6K demonstrates that this wire functions as part of an electrical circuit to light a light‐emitting diode (LED), confirming its utility in electronic applications.

## 3 Discussion and Conclusion

This study proposes an electrospray‐based SDREC strategy for the selective deposition of charged droplets and, in combination with CACM on silk fibroin, achieves programmable microstructure fabrication with a minimum feature size of 20 µm. Compared with existing microstructure array patterning methods, the approach offers several advantages: (i) printing of microstructures from ≈20 µm to centimeter scales; (ii) a programmable, erasable, and rewritable workflow enabled by a pixelated dot‐electrode architecture and the water solubility of silk fibroin; (iii) information encryption/decryption by exploiting the substantially higher haze of microstructured regions relative to flat regions together with the thermoresponsive behavior of SMP; and (iv) selective Ag deposition during the silver‐mirror (Tollens’) reaction by prepositioning Ag nanoparticles, yielding patterned silver electrodes.

As a proof of concept, the programmable array reported here is a 5×5 pixel matrix. The method is intrinsically scalable in both driver circuitry and pattern resolution; with established circuit‐driving and pattern‐design techniques, the pixel count and layout can be flexibly expanded to achieve higher resolution and more complex patterns, enabling practical applications in flexible electronics, information storage, and encryption.

## 4 Experimental Section

### 4.1 Materials

Quartz glass (JGS‐2, 1 mm thick) was purchased from Guluo Glass Co., Ltd. (China). PDMS (Sylgard 184) was obtained from Dow Corning (Shanghai) Co., Ltd. Rhodamine B (≥95%) was sourced from Tokyo Chemical Industry (TCI). Silver nanoparticles were purchased from XFNANO Materials Tech Co., Ltd. Silver nitrate (AgNO_3_) was obtained from Sinopharm Chemical Reagent Co., Ltd. Ammonia solution (3% NH_3_ in H_2_O) and glucose were purchased from Shanghai Macklin Biochemical Co., Ltd. Bisphenol‐A diglycidyl ether (BADGE) was obtained from Shanghai Acmec Biochemical Co., Ltd. Ethanol, acetic acid, sodium hydroxide, poly(propylene glycol) bis(2‐aminopropyl ether) (D‐230; polyetheramine), and decylamine (DA) were purchased from Aladdin Biochemical Technology Co., Ltd. 1H,1H,2H,2H‐perfluorooctyltriethoxysilane was also purchased from Aladdin. A 4 wt% silk fibroin solution was prepared from silkworm cocoons sourced from local farmers in Xi'an, China, as described in the literature [54]. Subsequently, the silk fibroin aqueous solution (4 wt%, 40 mL) was dialyzed at room temperature against a 20 wt% polyethylene glycol (PEG) (𝑀_n_ ≈ 20 000; Aladdin Biochemical Technology Co., Ltd.) solution using dialysis tubing with a 3.5 kDa molecular‐weight cutoff (MWCO). The volume ratio of PEG solution to silk fibroin solution was 30:1. After approximately 7 h, the concentrated silk fibroin solution (≈15 wt%) was slowly withdrawn with a syringe to avoid excessive shear. All reagents were used as received without further purification unless otherwise noted.

### 4.2 SDREC Panel Fabrication

Quartz‐glass substrates were ultrasonically cleaned in ethanol for 10 min and dried with nitrogen. The substrates were then treated with oxygen plasma in a plasma cleaner for 100 s to enhance surface cleanliness and wettability. AZ 4620 photoresist was spin coated in two steps: 500 rpm for 10 s followed by 2000 rpm for 40 s. The coated substrates were prebaked on a 95°C hotplate for 5 min. UV exposure was performed on a mask aligner for 20 s. After exposure, the patterns were developed in 0.5% (w/v) NaOH for 210 s, followed by a 5 min postbake at 95°C to harden the features. Metal films were deposited by magnetron sputtering: a 10 nm Cr adhesion layer followed by a 90 nm Au conductive layer. Lift‐off was carried out by immersing the samples in acetone with ultrasonication for 120 s, yielding Au‐patterned quartz substrates. Finally, a 15 wt% silk‐fibroin solution was spin coated onto the patterned Au electrodes at 500 rpm for 80 s to form the functional SDREC panel.

### 4.3 Fabrication of Patterned Microstructure Arrays

Patterned honeycomb‐like porous films were fabricated using the SDREC‐based patterned microstructure printing platform. All experiments were conducted under ambient laboratory conditions (temperature: 23–25°C, relative humidity: 40–50%), monitored using a thermohygrometer placed near the setup. Ethanol and deionized water were mixed at 15 wt%/85 wt%, respectively, and the mixture was allowed to stand for 48 h to remove entrained microbubbles and stabilize subsequent electrospraying. The solution was loaded into a 1 mL syringe mounted on a syringe pump. A 28G Musashi needle (inner diameter 180 µm) served as the nozzle and was connected to the positive terminal of a high‐voltage power supply. A grounded copper ring (inner diameter 14 mm, outer diameter 24 mm, thickness 2 mm) was positioned 3 mm below the needle tip. The SDREC panel was fixed on a motorized three‐axis translation stage; the designated metal features on the panel were grounded. The nozzle–substrate spacing was set to 10 mm. The flow rate was set to 200 µL h^−1^, and the applied voltage to 3.5 kV, yielding a stable electrospray. With the Z axis held fixed, the X–Y stage executed a raster fill trajectory to uniformly cover the entire panel area; three passes were performed at a scan speed of 1 mm s^−1^ with a line spacing of 1 mm.

### 4.4 Preparation of an SDREC Panel with Surface Fluorescence Distribution

Rhodamine B was dissolved in ethanol at a concentration of 0.1 mg mL^−1^. The solution was allowed to stand for 48 h to remove residual microbubbles and stabilize the electrospray. Ethanol was selected as the solvent because it does not dissolve silk fibroin. The Rhodamine B solution was loaded into a 1 mL syringe mounted on a syringe pump. A 28G Musashi needle (inner diameter 180 µm) served as the nozzle and was connected to the positive terminal of the high‐voltage power supply. The nozzle–substrate spacing was set to 10 mm. The copper ring and the designated metal features on the SDREC panel were grounded. The flow rate was adjusted to 100 µL h^−1^, and the applied voltage to 2.3 kV, yielding a stable electrospray. With the Z axis held fixed, the X–Y stage executed a raster fill trajectory to uniformly cover the entire panel area in one pass at a scan speed of 1 mm s^−1^ with a line spacing of 1 mm.

### 4.5 Uniform Surface Charging by Electrospraying Pure Ethanol

Ethanol was electrosprayed onto the SDREC panel under the same setup and parameters (28G needle, 10 mm spacing, 100 µL h^−1^, 2.3 kV). The copper ring was grounded, while the patterned electrodes were kept ungrounded. A raster‐fill scan was performed to cover the entire panel area, and the scan was repeated twice under identical conditions.

### 4.6 Finite‐Element Electrostatic Simulation

Electrostatic simulations were performed using a finite‐element model (COMSOL Multiphysics, Electrostatics module) constructed according to the experimental stack. From bottom to top, the model consisted of a 1‐mm‐thick quartz substrate, patterned metal electrodes, and a 3‐µm‐thick silk fibroin layer. The relative permittivities were set to ε_r_(quartz) = 3.8 and ε_r_(silk fibroin) = 6.1. A uniform surface charge density of σ = 1 × 10^−^^5^ C m^−^^2^ was applied on the top surface of the silk fibroin layer to represent the deposited residual charges. Boundary conditions were applied as follows: the bottom surface of the quartz substrate was grounded. The patterned electrodes were assigned either grounded or floating conditions, depending on the SDREC state being modeled.

### 4.7 Preparation of SMP for Information Encryption (Including Encryption/Decryption Procedure)

A PDMS positive (protruding) mold was first obtained by pouring a degassed PDMS prepolymer mixture (10:1 w/w, Sylgard 184, Dow Corning) onto the patterned honeycomb‐like porous film, curing at 65°C for 4 h, and peeling off the cured elastomer. For fluorosilane treatment, a solution of 1H,1H,2H,2H‐perfluorooctyltriethoxysilane (FOTS) in ethanol (2 wt%) was prepared, followed by the addition of 1–2 wt% water and 0.5 wt% acetic acid; the mixture was stirred for 5 h to allow hydrolysis. The PDMS positive mold was immersed in the FOTS solution for 20 min and then air‐dried at ambient conditions to reduce adhesion and facilitate demolding. A PDMS porous (negative) mold was then prepared by pouring the degassed PDMS prepolymer onto the silanized PDMS positive mold, curing at 60°C for 4 h, and peeling to obtain the negative relief. The SMP was formulated following a literature‐reported recipe [55]. Briefly, BADGE, Jeffamine D‐230, and decylamine (DA) were mixed at a 4:1:2 molar ratio, degassed by ultrasonication for 1 h, cast into the PDMS porous mold, and thermally cured at 100°C for 1.5 h and then 130°C for 1 h. After cooling to room temperature, the cured SMP was carefully demolded from the PDMS mold. For information encryption/decryption, the SMP replica was heated on a hot plate to 80°C. Encryption (programming) was performed by applying a compressive load to flatten the surface microstructures, followed by cooling under load to fix the temporary state in which the encoded pattern became optically indistinguishable. Decryption (recovery) was achieved by reheating the sample to 80°C without external load, allowing the microstructures to recover and the encoded pattern to reappear.

### 4.8 Fabrication of Patterned Silver Electrodes

A 1 mL syringe containing the silver colloid was mounted on a syringe pump. A 28G Musashi needle (inner diameter 180 µm) served as the nozzle and was connected to the positive terminal of a high‐voltage supply. The nozzle–substrate spacing was set to 10 mm. The copper ring and the designated metal features on the SDREC panel bearing the patterned honeycomb‐like microstructures were grounded. The flow rate was adjusted to 100 µL h^−1^, and the applied voltage to 2.3 kV, yielding a stable electrospray. With the Z axis held fixed, the motorized X–Y stage executed a raster fill trajectory to uniformly cover the entire panel in one pass at 1 mm s^−1^ with a 1 mm line spacing. To transfer the deposited nanoparticles, degassed PDMS prepolymer (10:1 w/w, Sylgard 184) was poured over the sprayed sample, cured at 65°C for 4 h, and peeled off to obtain a PDMS replica bearing surface Ag nanoparticles. For fluorescent labeling, rhodamine B was dissolved in the silver colloid at 0.1 mg mL^−1^ and processed identically to yield a PDMS replica with fluorescently tagged surface Ag nanoparticles. Tollens’ reagent was freshly prepared as follows: 10 mL of 2 wt% AgNO_3_ aqueous solution was mixed with 10 drops of 5 wt% NaOH to form a precipitate. Subsequently, 3 wt% aqueous ammonia was added dropwise with gentle shaking until the precipitate just dissolved, resulting in a clear Tollens’ solution. For selective silver growth (silver‐mirror reaction), a fresh mixture of 1.5 mL glucose aqueous solution (10 wt%) and 3 mL Tollens reagent was drop‐cast onto the Ag‐nanoparticle–seeded PDMS surface. Prior to mixing, both the glucose solution and the Tollens reagent were held at 50°C in a water bath. After the mixture spread across the patterned area, the reaction proceeded at 60°C for 1–3 min. The sample was then rinsed with deionized water to remove residues and dried prior to use.

### 4.9 Characterization

Fluorescence distribution images were acquired using a super‐resolution confocal microscope (TCS SP8 STED 3X, Leica Microsystems, Germany). Surface potential measurements were performed on an atomic force microscope (AFM) (Bruker Dimension XR, Bruker, USA) operated in PeakForce KPFM mode with an SCM‐PIT‐V2 probe. Surface micro‐/morphological features were examined by field‐emission scanning electron microscopy (SU‐8010, Hitachi High‐Tech, Japan). Haze measurements were performed on a UV–Vis–NIR spectrophotometer (UV‐3600, Shimadzu, Japan), over the wavelength range of 380–780 nm. The electrical performance of the printed silver electrodes was evaluated with a four‐point‐probe DC low‐resistance meter (FP‐001, Kaivo, China).

## Funding

The Research and Development Program of Shaanxi Province, China [grant number 2021ZDLGY10‐09]; The High‐End Foreign Expert Recruitment Program, China [grant number G2023170009L]; The Science and Technology on Electromechanical Dynamic Control Laboratory, China [grant number No. 6142601220111]

## Conflicts of Interest

The authors declare no conflicts of interest.

## Supporting information




**Supporting file**: advs74488‐sup‐0001‐SuppMat.pdf.

**Supporting Movie**: advs74488‐sup‐0002‐Movie S1.Erasure of Microstructured Patterns.mp4.

**Supporting Movie**: advs74488‐sup‐0003‐Movie S2.mp4.

**Supporting Movie**: advs74488‐sup‐0004‐Movie S3.Information Decryption Pattern Reappears on SMP.mp4.

**Supporting Movie**: advs74488‐sup‐0005‐Movie S4.QR Code Reading After Decryption.mp4.

## Data Availability

All data are available in the main text or the Supporting Information.
