# Supplementary material for: Selective Deposition of Charged Droplets for Programmable and Rewritable Printing of Patterned Microstructure Arrays
Source: Adv Sci (Weinh). 2026 Feb 24;13(25):e22210. doi: 10.1002/advs.202522210 (PMC13137844; doi:10.1002/advs.202522210)
Supplement: Supplementary file 1 — Supporting file: advs74488‐sup‐0001‐SuppMat.pdf. [file ADVS-13-e22210-s002.pdf]

Supporting Information

Selective Deposition of Charged Droplets for Programmable and Rewritable Printing of Patterned Microstructure Arrays

*Yuechen Pei, Li Wang *, Zhaofa Zhang, Yulin Zheng, Tianxue Yin, Jiaye Chen, Juntao Zhang, Yujie Qi, Bingheng Lu*

Supplementary Note

The detailed derivation of the electric field induced by residual charges

Consider a system composed of multiple dielectric and conductor layers, where dielectric 2 and the metal layer have lateral dimensions much larger than their thickness. The structure, from bottom to top, is as follows:

Dielectric 1 (quartz glass substrate): Dielectric constant = ε_1.

Metal conductor plane (gold electrode layer).

Dielectric 2 (silk fibroin film): Dielectric constant = ε_2.

Residual charges: Uniformly distributed surface charge with surface density σ on the upper surface of silk fibroin film.

The remaining space is air with dielectric constant ε.

We aim to determine the distribution of the electric field intensity in the region above the system (i.e., in the air above silk fibroin film) under electrostatic equilibrium and neglecting edge effects, for the following two cases:

Case 1: The metal conductor is not grounded, with net charge equal to zero.

Case 2: The metal conductor is grounded.

Case 1: Metal Conductor Not Grounded, Net Charge = 0

At electrostatic equilibrium, no net charge resides inside the conductor, so charges are distributed only on the upper and lower surfaces of the metal conductor. Neglecting edge effects, these induced charges can be considered uniformly distributed. Let σ_1 denote the induced surface charge density on the upper surface, and σ_2 the induced surface charge density on the lower surface.

By the law of charge conservation:

$$\sigma_1 + \sigma_2 = 0$$

As shown in the **Figure S1A**, we construct Gaussian surfaces S_1, S_2, and S_3. Using Gauss's law in the presence of dielectrics, we obtain:

$$\varepsilon E_{\text{I}} - \varepsilon_2 E_{\text{II}} = \sigma$$

$$\varepsilon_2 E_{\text{II}} = \sigma_1$$

$$\varepsilon_1 E_{\text{IV}} = \sigma_2$$

The electric field at a point P inside the metal layer is the superposition of the fields from the three charged surfaces, hence:

$$E_P = \frac{\sigma}{2\varepsilon} + \frac{\sigma_1}{2\varepsilon} - \frac{\sigma_2}{2\varepsilon}$$

Since the electric field inside a conductor at electrostatic equilibrium is zero, $E_p = 0$, therefore:

$$\sigma + \sigma_1 - \sigma_2 = 0$$

By combining the above expressions, we obtain:

$$\sigma_1 = -\frac{\sigma}{2}, \sigma_2 = \frac{\sigma}{2}$$

$$E_{\text{I}} = \frac{\sigma}{2\varepsilon}$$

Case 2: Metal Conductor Grounded

Let σ_1 and σ_2 again denote the induced surface charge densities on the upper and lower surfaces of the metal conductor, respectively. When the metal conductor is grounded, it becomes an equipotential body connected to the Earth (a large conductor). The charges on its

lower surface are dispersed over the much larger surface area of the Earth, effectively eliminating any charges on the lower surface. Therefore:

$$\sigma_2 = 0$$

Similar to Case 1, we construct Gaussian surfaces S_1, S_2, and S_3 (see **Figure S1B**). Applying Gauss's law for dielectrics yields:

$$\varepsilon E_{\mathrm{I}} - \varepsilon_2 E_{\mathrm{II}} = \sigma$$

$$\varepsilon_2 E_{\mathrm{II}} = \sigma_1$$

$$\varepsilon_1 E_{\mathrm{IV}} = \sigma_2$$

For the electric field at point P inside the conductor to be zero, the following must hold:

$$\sigma + \sigma_1 = 0$$

By combining the above expressions, we obtain:

$$\sigma_1 = -\sigma$$

$$E_{\mathrm{I}} = 0$$

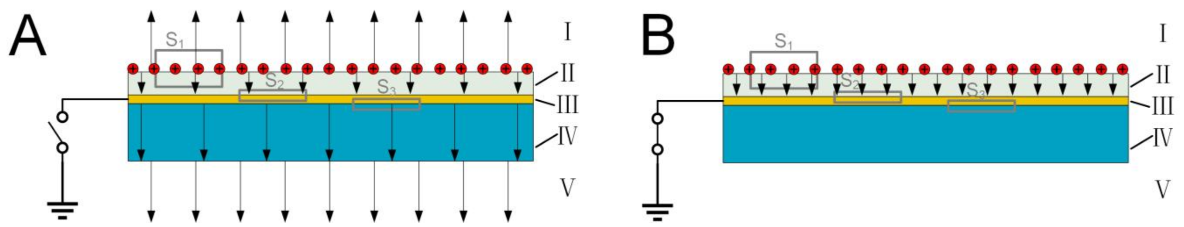

Figure S1. Diagram of the Gaussian surface and electric field distribution.

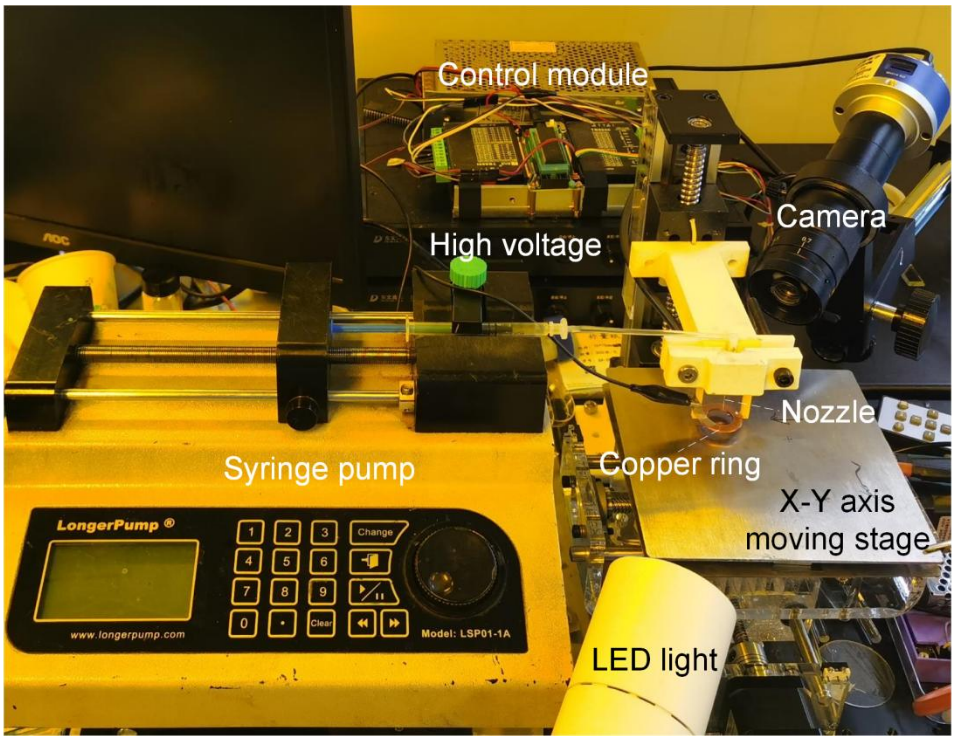

Figure S2. Photograph of a patterned microstructure printing system based on SDREC.

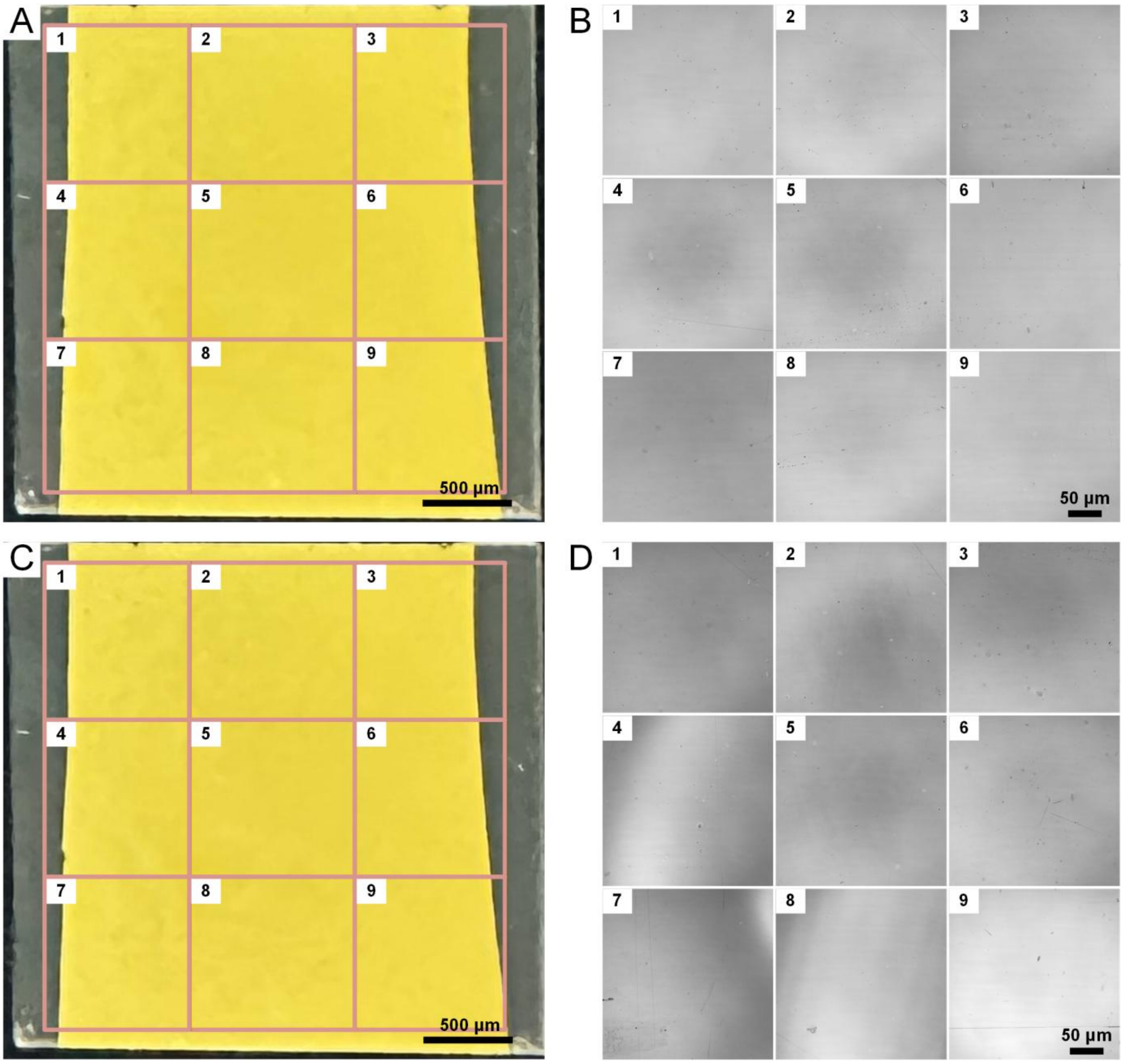

Figure S3. Morphological assessment of DI-water erasure on the SDREC panel surface. (A) Photograph/schematic of the SDREC panel divided into nine regions (1–9). (B) 3×3 montage of laser confocal microscopy surface maps acquired from one randomly selected location in each region before DI-water erasure (maps labeled 1–9); the corresponding areal roughness values Sa are 0.01, 0.02, 0.024, 0.009, 0.023, 0.023, 0.018, 0.021, 0.010 µm. (C) Optical image of the panel after DI-water erasure. (D) 3×3 montage of confocal surface maps acquired from one randomly selected location in each region after erasure (maps labeled 1–9); the corresponding Sa values are 0.029, 0.019, 0.027, 0.023, 0.026, 0.023, 0.018, 0.021, 0.013 µm. Mean ± s.d.: 0.0176 ± 0.0062 µm (before) and 0.0221 ± 0.0050 µm (after), n = 9.

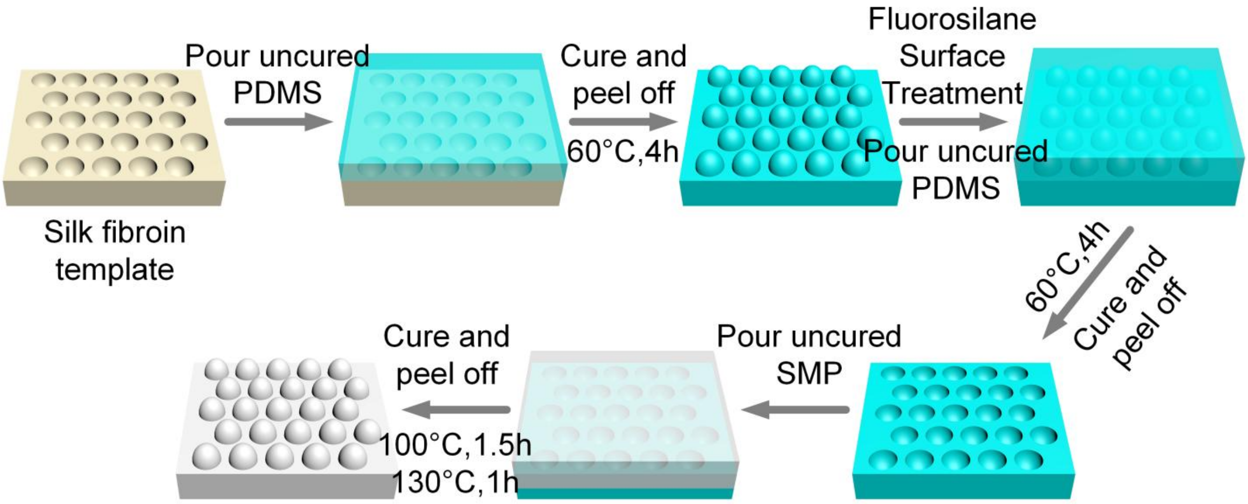

Figure S4. Transfer of SDREC-patterned microstructures to an SMP via two-step replica molding.
Starting from an SDREC-patterned silk-fibroin template with concave features, uncured PDMS is poured, cured (60 °C, 4 h), and peeled to obtain a PDMS positive (protruding) replica; the PDMS positive is then fluorosilane-treated to reduce adhesion, after which a second layer of uncured PDMS is cast, cured (60 °C, 4 h), and peeled to yield a PDMS negative (concave) mold; uncured SMP is poured onto the PDMS negative, thermally cured (100 °C, 1.5 h; then 130 °C, 1 h), and peeled off to produce an SMP positive relief that faithfully reproduces the original microstructures.

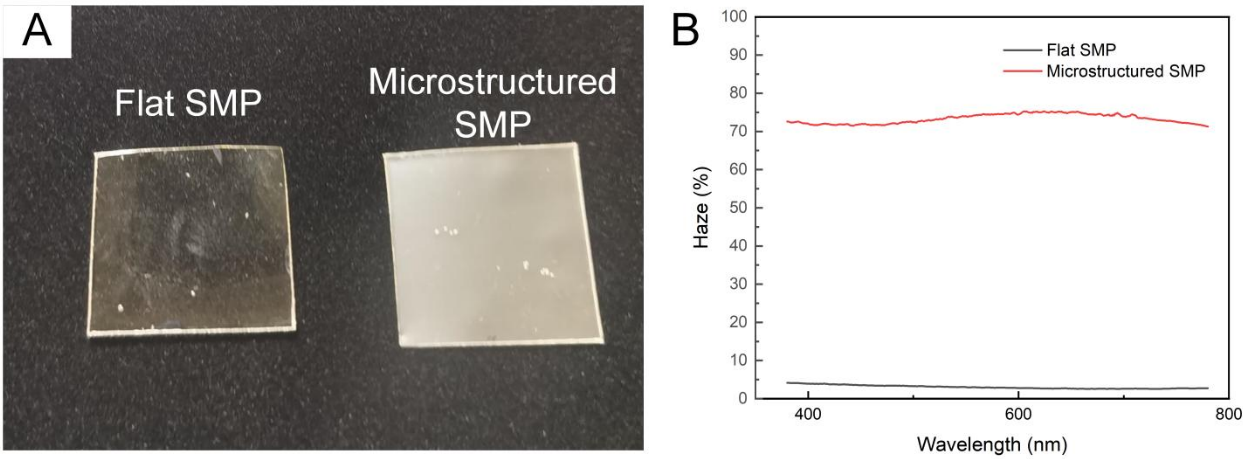

Figure S5. Optical appearance and haze of SMP with and without surface microstructures.
(A) Photographs of a flat SMP (transparent) and a microstructured SMP (visibly white due to microstructure-induced scattering). (B) Haze versus wavelength in the visible band (380–780 nm): the microstructured SMP shows strong scattering with a maximum haze of 75.3%, whereas the flat SMP remains nearly transparent with a maximum haze of 4.19%. The large haze contrast enables clear visual readout of patterned regions against a transparent background.

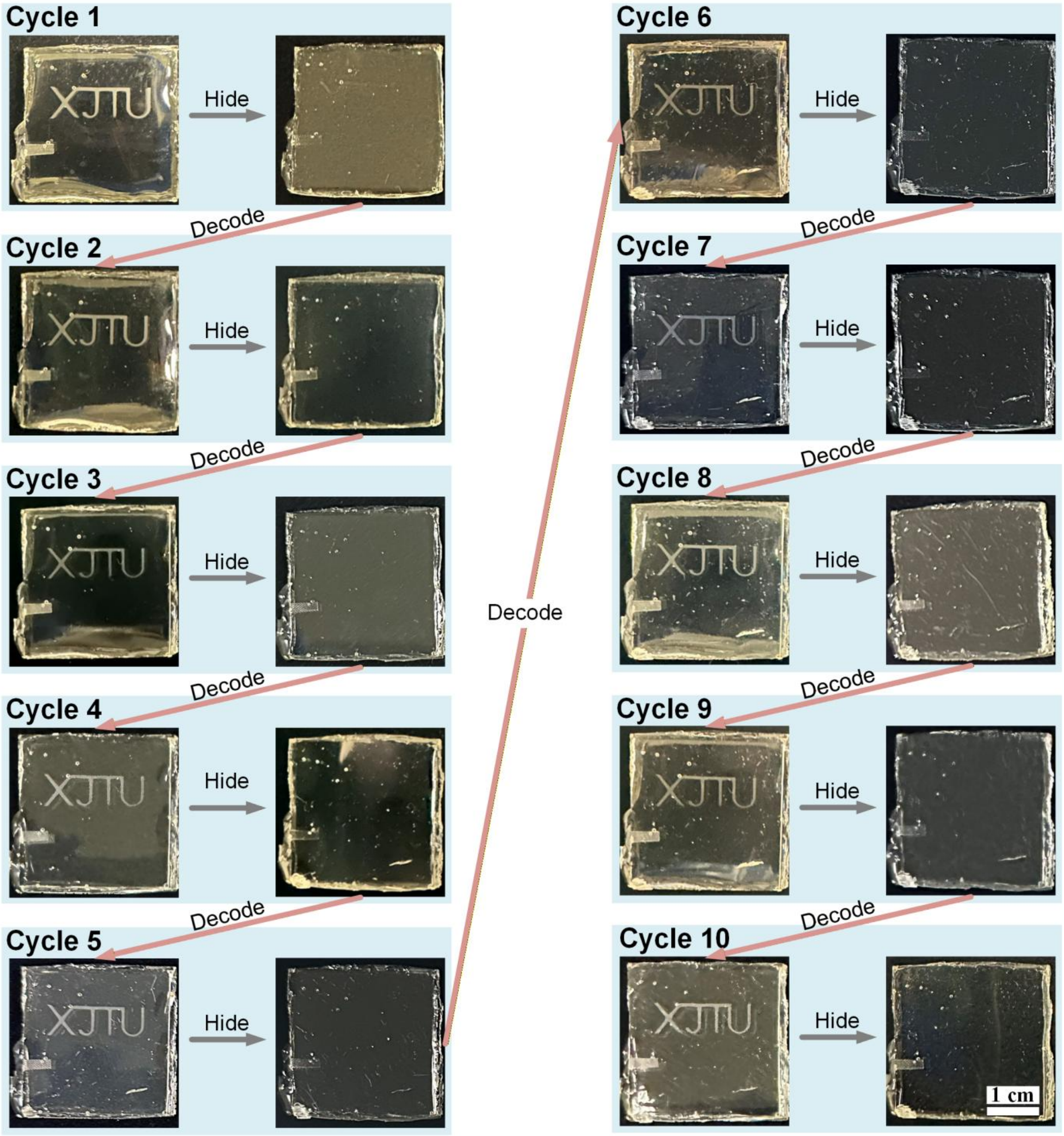

Figure S6. Cycling stability of SMP-based optical encryption/decryption (optical images). Optical photographs recorded during 10 consecutive encryption/decryption cycles of the SMP replica. For each cycle, the sample was decrypted by heating at 80 °C, where the “XJTU” pattern became clearly visible, and then encrypted by heating at 80 °C, applying pressure to flatten the surface microstructures, and cooling under pressure to fix the temporary state, where the pattern became invisible. The pattern remains repeatable and readable after at least 10 cycles.

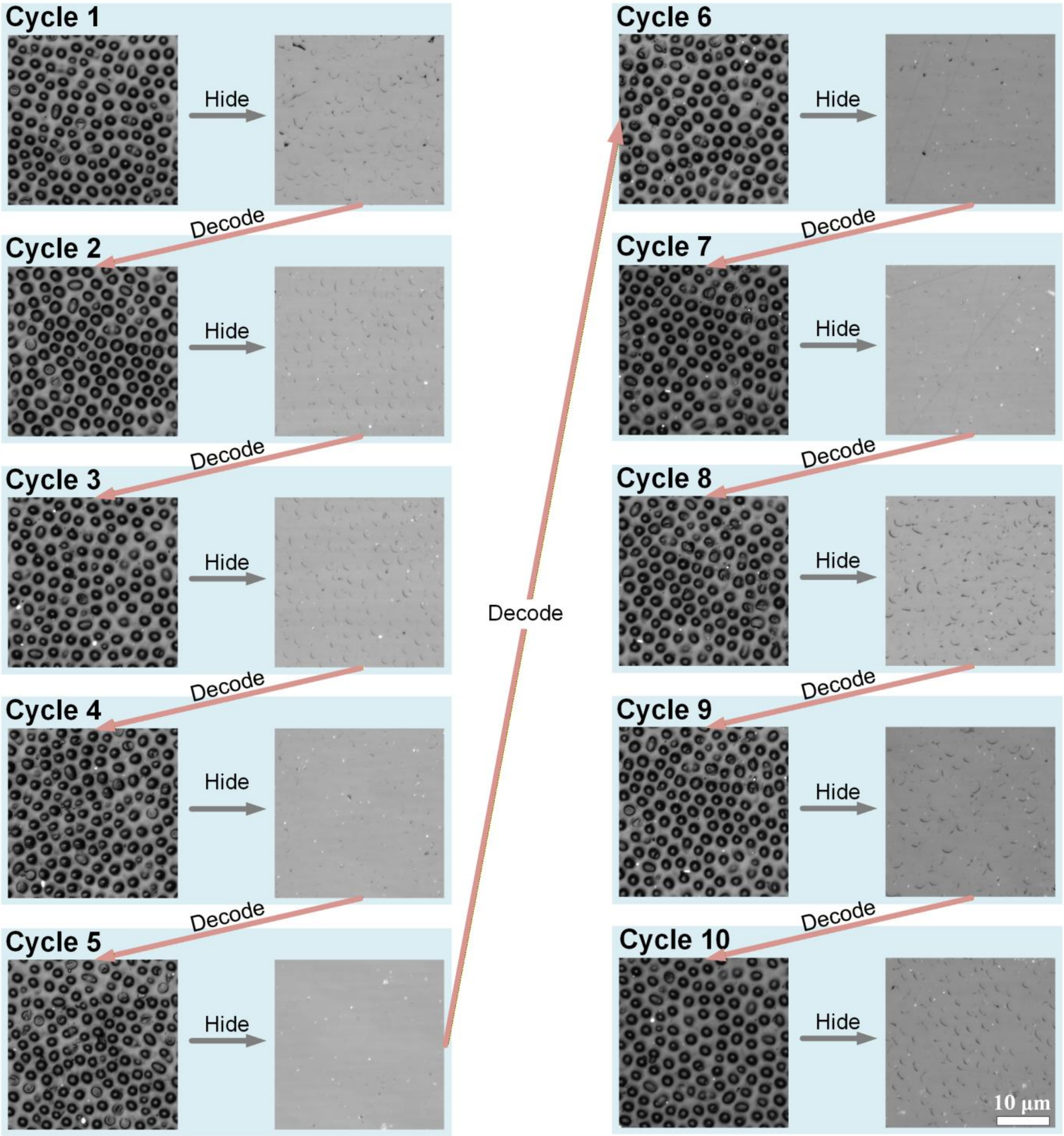

Figure S7. Cycling stability of SMP-based optical encryption/decryption (laser confocal microscopy).
Laser confocal microscopy images acquired in every cycle to track the reversible microstructure switching. In the decrypted state, the surface microstructures are clearly resolved; in the encrypted state (heated to 80 °C, pressed to flatten, and cooled under pressure), the microstructures are flattened. The morphology and switching behavior remain consistent over 10 cycles, indicating no observable structural damage under the tested conditions.

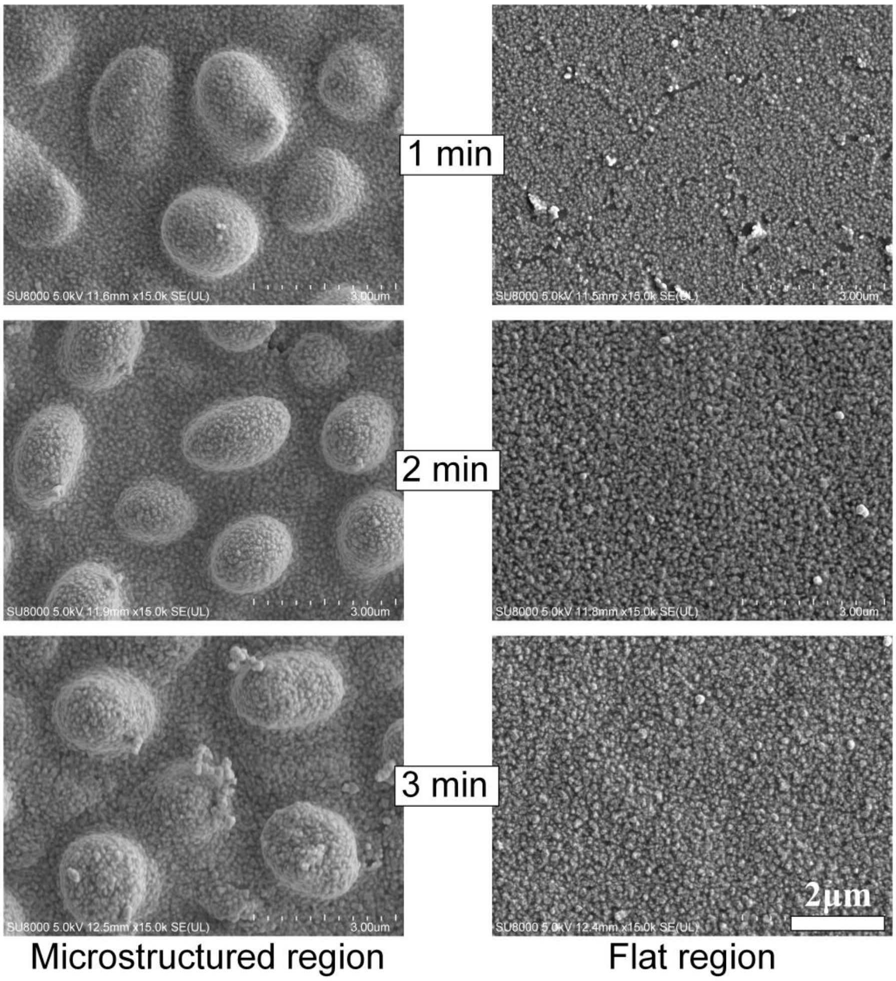

Figure S8. SEM morphology of silver on PDMS after the silver-mirror reaction at different regions and times.
SEM images of the PDMS surface after silvering, comparing microstructured and flat regions (columns) at reaction times of 1, 2, and 3 min (rows). In the absence of Ag-nanoparticle seeding, silver deposits uniformly across both regions at all times.

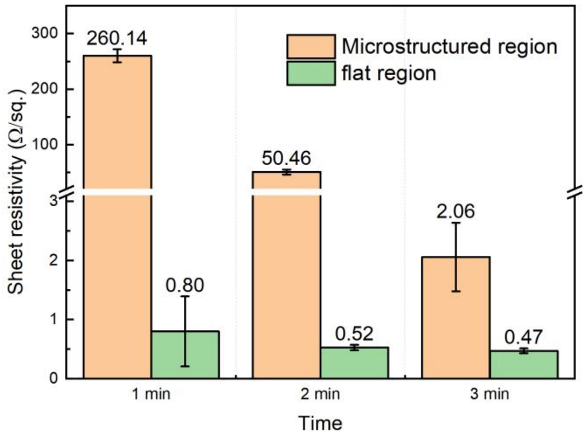

Figure S9. Electrical properties corresponding to Figure S4 (control, no Ag-nanoparticle seeding).
Bar chart of sheet resistance (ohms/sq; four-point probe) for samples, comparing the microstructured (orange) and flat (green) regions at 1, 2, and 3 min reaction times. Both regions are conductive at all times; the microstructured region decreases from ~260.14 to ~2.06 ohms/sq as silver coalesces, while the flat region remains low (~0.80, 0.52, 0.47 ohms/sq). Error bars denote standard deviation.

Table S1. Comparison of SDREC with representative patterning techniques.

Method	Structure feature size (unit microstructure)	Pattern feature size (minimum patterned feature)	Mask / mold requirement	Cleanroom requirement	Reconfigurable during fabrication	Erasable / rewritable after fabrication	Equipment / infrastructure cost level	Ref.
**SDREC + CACM**	0.9–3.4 µm	20 µm	No	No	Yes	Yes	Low–Medium	This work, [35]
**Photolithography**	sub-µm	sub-µm	Yes (photomask)	Yes	Limited (new mask required)	No	High	[27,28]
**Electron-beam lithography**	<10 nm	<10 nm	No	Yes	Yes	No	Very high	[29-31]
**Nanoimprint lithography**	nm-scale (mold-limited)	nm-scale (mold-limited)	Yes (imprint mold/stamp)	No	Limited (new mold required)	No	Medium–High	[32-34]
